# Antibacterial effect of ozonated water against methicillin-resistant *Staphylococcus aureus* contaminating chicken meat in Wasit Province, Iraq

**DOI:** 10.14202/vetworld.2018.1445-1453

**Published:** 2018-10-18

**Authors:** Manal H. G. Kanaan

**Affiliations:** Department of Nursing, Technical Institute of Suwaria, Middle Technical University, Baghdad, Iraq

**Keywords:** antibacterial effect, chicken meat, methicillin-resistant *Staphylococcus aureus*, ozonated water, Wasit Province

## Abstract

**Background and Aim:**

Methicillin-resistant *Staphylococcus aureus* (MRSA) is one of the most recognized “superbugs” and a common cause of community-associated and nosocomial infections; furthermore, when chicken meat is considered a good growth medium for *S. aureus* to make a plausible vehicle to propagate MRSA, then this study was conducted to evaluate the efficiency of ozonated water (0.5 ppm) in the elimination or reduction of MRSA contaminating fresh and frozen chicken meat sold in local markets in the Wasit Province.

**Materials and Methods:**

A total of 72 samples of fresh and frozen chicken meat were randomly collected from dissimilar native markets: Fresh chicken meat (n=32) and frozen chicken meat (n=40). Isolation and identification of MRSA isolates were conducted using standard bacteriological, biochemical, RapID™ Staph Plus System (Remel, R8311009), and latex agglutination tests such as Dry SPOT Staphytect Plus (Oxoid, DR0100M) and PBP2’ Test Kit (Oxoid, DR0900A). The generation of ozone (O_3_) was carried out using O_3_ generator (A2Z/AQUA-6, USA), and its concentration (ppm) in water was determined using CHE-Mets^®^-Kit, USA.

**Results:**

A total of 39 (54.2%) of 72 fresh and frozen chicken meat were positive for *S. aureus*; of those 39 positive samples, 13 (33.3%) were identified as MRSA. The antibiotic sensitivity test results revealed that all MRSA isolates had multiple resistance to at least four antimicrobial agents for which these isolates had 12 antibiotic resistance patterns. Results of O_3_ treatment in MRSA isolate contaminating 13 of both fresh and frozen chicken meat samples showed that, after treatment with ozonated water (0.5 ppm/4°C), the overall negative samples were 23.1% and 69.2% for 30 and 45 min, respectively. The decrease in the percentage of positive samples was very significant from a public health perspective. Furthermore, the antimicrobial efficacy of ozonated water (0.5 ppm) on the reduction of the MRSA count (log_10_ colony-forming units [CFU]/ml) was assessed in four positive samples of fresh and frozen chicken meat, and the results revealed that, after treatments, the overall reduction was 2-4 log_10_ (CFU/ml) after 45 min. This reduction is highly significant from a public health perspective.

**Conclusion:**

From the data obtained from this study, it can be concluded that fresh and frozen chicken meat sold in the different markets of Wasit Province was highly contaminated by *S. aureus* during the study period with a total prevalence of 54.2%; among those, 33.3% were recognized as MRSA. Under the conditions described in the present study, O_3_ at the concentration of 0.5 ppm is highly effective in reducing the number of MRSA-positive samples and the number decreased with increased exposure time to ozonated water at the same concentration. These findings indicated that O_3_ treatment might constitute the basis for an alternative method to reduce meat contamination with foodborne pathogens such as MRSA.

## Introduction

*Staphylococcus aureus* is an illustrious opportunistic foodborne pathogen and is involved in numerous nosocomial and community-associated outbreaks worldwide [1]. It is a dangerous bacterium considering its negative consequences for animal health and its ability to transmit from animals to people and *vice versa* [2]. In addition, toxigenic foodborne strains of *S. aureus* also pose a threat to humans who ingest food contaminated with preformed enterotoxins [3]. The strains of *S. aureus* transmitted by food tend to be resistant to different types of antibiotics [4]. Today, methicillin-resistant *S. aureus* (MRSA) has become a serious health problem due to its high resistance to different types of antibiotics [5]. MRSA has been found in several meat-producing animals throughout the world, including pigs, chickens, and cattle [6]. The prevalence of livestock-related MRSA in farm animals is increasing, and the resulting foods may be contaminated [7]. On the other hand, the appearance of ST398 in bovine [8], CC398 and CC5 in poultry [9], and the probability of these strains to cause serious infections and even death in humans [10] demonstrate the importance to investigate meat products such as poultry meat as possible vehicles for the transmission of MRSA at the consumer level [8]. Worldwide, consumers prefer chicken meat, especially in recent years due to its low-fat content, excellent quality protein, quick preparation, and cost-effectiveness compared to red meat [11]. The contamination of chicken meat by pathogenic and harmful bacteria occurs mainly during scalding, plucking, and evisceration of chicken carcasses. In addition, cross-contamination of processing water and equipment increase the opportunity for contamination in the processing stages [12]. Therefore, to eliminate bacterial contaminants in chicken meat, numerous processing techniques have been developed in the food industry, including chlorine, high-pressure processing, gamma radiation, ultraviolet radiation, and ozone (O_3_) [13]. Chlorine is widely used in the sanitation of poultry operations, and the use of chlorine is more scrutinized due to the problems of toxicity and disinfection byproducts that have proven to be harmful to food safety and from the environmental point of view [14].

Researchers in the food industry are trying to find alternative cleaning and disinfecting agents that are effective against spoilage of food and pathogenic bacteria that are harmless to humans and the environment. These investigations of chlorine alternatives in poultry operations, particularly in the chiller, are of interest to the poultry industry [12]. O_3_ is the natural substance in the atmosphere that has involved the attention of food scientists as an alternative disinfectant [15]. This gas can be applied in the food industry for bacterial elimination, inactivation of viruses, and others [16,17]. Even if it does not leave harmful residues in the treated products, it decomposes rapidly into oxygen [18]. The use of O_3_ increased after its designation as generally recognized as safe by the Food and Drug Administration in 1997 [19]. It has been reported that O_3_ eliminates foodborne pathogens such as *Salmonella*, *Listeria monocytogenes*, and *S. aureus* [20]. Therefore, O_3_ is considered the most appropriate for the elimination of microbes of food safety problems [21].

In Iraq, chicken meat is considered the most popular meat in many communities, and due to the absence of data on the prevalence of MRSA in chicken meat, this study was conducted to investigate the level of contamination of chicken meat with MRSA that would help in the evaluation of the effect of O_3_ treatment on the microbiological safety of chicken meat sold in Wasit markets.

## Materials and Methods

### Ethical approval

Ethical approval is not necessary to pursue such type of study. Meat samples were collected from markets.

### Collection and processing of samples

From November 2017 to February 2018, a total of 72 samples of chicken meat, including fresh chicken meat (n=32), locally produced frozen chicken meat (n=20), and imported frozen chicken meat (n=20), were collected at random from several supermarkets and retailer stores. The samples were packaged in a sterilized polyethylene bag and sent to the laboratory with ice packs in 3 h. The chicken samples were divided into two parts: The first portion was subjected to microbiological evaluation and the second portion was kept in a freezer at −18°C for further analysis.

### Isolation and identification of S. aureus and MRSA

The samples were analyzed and processed according to standard food microbiological procedures [22-24], with some modification. A portion of 10 g was cut and transferred into the sterilized plastic bag, then supplemented with 90 ml of buffered peptone water (BPW) (Oxoid, CM0509), and homogenized in a stomacher for 3 min. About 5 ml of the homogenate was removed into 50 ml of Tryptone Soya Broth (Oxoid, CM0129) with 0.6% yeast extract (YE). After incubation at 35°C for 18 h, 20 µL of the culture was plated onto Baird-Parker agar (Oxoid, CM1127) supplemented by Egg Yolk Tellurite and incubated overnight at 37°C. Colonies exhibiting typical morphological characteristic of MRSA on Baird-Parker agar (bright black colonies surrounded by 2-4 mm clear zones after 48 h) were purified on Baird-Parker agar without supplement at 35°C for 24 h and preserved on Tryptic Soy Agar-YE at 4°C with 0.02% of thiomersal solution [25].Further identification based on Gram-staining, fermentation of mannitol salt agar (Oxoid, CM0085), activity of blood hemolysis on horse blood agar (Oxoid, CM0854), catalase activity, coagulase test (rabbit and human plasma) was done [22-26]. In addition, the RapID™ Staph Plus System (Remel, R8311009) was used to confirm the identification of *S. aureus* at the species level. The isolates of MRSA were identified using SPOT Staphytect Plus (Oxoid, DR0100M), which is a latex agglutination test for the detection of aggregation factor, protein A, and some polysaccharides found in MRSA isolates [27]. The isolates were identified another time using PBP2’ Test Kit (Oxoid, DR0900A), which is a latex agglutination test for the rapid detection of PBP2a in the *S. aureus* isolates whereby agglutination was observed within 3 min indicating PBP2a positive (MRSA) [27].

### Antibiotic sensitivity test of MRSA isolates

A Kirby–Bauer disk diffusion technique on Mueller-Hinton agar (Oxoid, CM0337) according to the Clinical and Laboratory Standards Institute [28] was adopted to assess the antimicrobial susceptibility of the MRSA isolates against 10 antimicrobial agents: ME (5 μg), cefoxitin (FOX) 30 μg, oxacillin (OX) 1 μg, gentamicin (GM) 10 μg, ofloxacin 5 μg, erythromycin (E) 15 μg, tetracycline (T) 30 μg, enrofloxacin (ENF) 5 μg, vancomycin (VAN) 30 μg, and fusidic acid (FA) 10 μg (Oxoid, UK).

### Calculation of O_3_ concentration production (ppm/in water)

The O_3_ concentration (ppm) in water produced by the O_3_ generator (A2Z/AQUA - 6 Specifications) was carried out using O_3_ CHE-Mets^®^-Kit and as a technique implemented by Abdulateef [29]. Briefly, a plastic container was occupied with 1.5 L of tap water and enclosed by its cover. The aeration stone was injected into the container through a hole in the cover. 5 exposure times (contact times) were chosen (5, 10, 15, 30, and 45 min). After each exposure time, the tap water was altered, and the container flushed with new tap water, and the process was recurrent. The empty sample cup was supplemented with five droplets of A-7400 Activator and then filled to the 25 ml mark with the ozonated water, the CHEMet ampoule tip was located into the cup, and the tip of the ampoule was broken and occupied by the ozonated water, then inverted several times, and left for 1 min for color change. The ampoule was placed between the color standards until the best color was matched by high range comparator [29]. The highest concentration among the 5 times used was got at 15, 30, and 45 min, which was 0.5 ppm/in water.

### The effect of ozonated water (0.5 ppm) on MRSA

The second portion of each positive sample of chicken meat (from the first step) was exposed to the second step. In this test, O_3_ gas was injected into the water using aeration stone (Diffuser) and spread evenly through the water. The O_3_ generator was supplied with 1 L/min (600 mg/h) of compressed air as a feed gas. The samples were deiced in a refrigerator overnight and then submerged into ozonated water at 4°C. The ozonated water was spread within the chicken meat samples for 2 different experience times (30 and 45 min). A portion of 10 g of each sample was chopped and moved into a sterile plastic bag and treated as described previously. The bactericidal effect of O_3_ was achieved using the method of Miles and Misra [30], through estimating the number of colony-forming units (CFU) in a bacterial suspension in which a sequence of decimal 10 dilutions of enrichment broths was diluted with sterile BPW tubes (1 ml broth/9 ml BPW), and then, 5 drops×20 μl of each dilution sequence was let fall onto Baird-Parker agar and let dry before incubation at 37°C for 24 h. The microbial load log titer was calculated as follows:

CFU per ml=Average number of colonies for a dilution×50×dilution factor [30].

### Statistical analysis

The effect of the variables (antibiotics) on MRSA isolates based on chicken source was determined using Chi-square test through Statistical Package for the Social Sciences ver. 18.0 (IBM, USA). The significant differences among variables (p-value) in any test were as follows:

S=Significant difference (p<0.05).

NS=Non-significant difference (p>0.05).

## Results

In this study, the prevalence of *S. aureus* isolates in fresh and frozen chicken meat samples was scrutinized. The results (Table-1) presented that 39 (54.2%) of 72 were positive for *S. aureus* by which the prevalence of *S. aureus* isolates in fresh and frozen chicken meat was 59.4% and 50%, respectively. Imported chicken meat displayed the lowest prevalence (45%), while fresh chicken meat had the highest prevalence (59.4%). Furthermore, the results also revealed that 13 (33.3%) of 39 *S. aureus* isolates were recognized as MRSA with isolation percentages of 42.11% and 25% in fresh and frozen chicken meat, respectively.

**Table-1:** Prevalence of *S. aureus* and MRSA in fresh and frozen chicken meat retailed in Wasit Province.

Sample type	Source	Number of samples tested	n/N (%) of *S. aureus*-positive samples	n/N (%) of MRSA-positive samples
Fresh chicken meat	_	32	19/32 (59.4)	8/19 (42.11)
Frozen chicken meat	Local	20	11/20 (55)	3/11 (27.3)
	Imported	20	9/20 (45)	2/9 (22.2)
	Total (frozen)	40	20/40 (50)	5/20 (25)
Total	_	72	39/72 (54.2)	13/39 (33.3)

*S. aureus=Staphylococcus aureus*, MRSA=Methicillin-resistant *Staphylococcus aureus*

**Figure-1:**
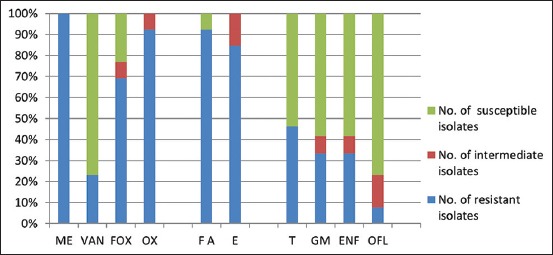
Prevalence of antimicrobial resistance in methicillin-resistant *Staphylococcus aureus* isolates recovered from fresh and frozen chicken meat.

Antibiotic resistance patterns (ARP) of 13 MRSA isolates recovered from fresh and frozen chicken meat samples were scrutinized, and the results are shown in Figure-1. The results revealed that MRSA isolates presented high prevalence of resistance against ME (100%), OX (92.3%), FA (92.3%), E (84.6%), and FOX (69.2%) and low prevalence of resistance against T (46.2%), ENF (38.5%), GM (30.8%), VAN (23.07%), and OFL (7.7%). Data analysis revealed that there were significant differences in the levels of resistance by chicken source only seen with FOX (p=0.05) and VAN (p=0.01) (Table-2). The acquired results (Table-3) revealed that all MRSA isolates (100%) exhibited multidrug resistance (MDR) to at least four antimicrobials by which these isolates displayed 12 ARPs (Table-3). Although no pattern was common between fresh and frozen chicken meat, two isolates (one recovered from fresh and another recovered from frozen chicken) were resistant to nine antimicrobials, with which the fresh MRSA isolate offered resistance to six classes of antimicrobials and exhibited the MDR model of ME, FOX, OX, FA, E, T, GM, ENF, and OFL, while the frozen MRSA isolate offered resistance to seven classes of antimicrobials and exhibited the MDR model of ME, VAN, FOX, OX, FA, E, T, GM, and ENF.

**Table-2:** Data analysis of antibiotic sensitivity test for MRSA isolates based on sample sources.

Antibiotics	Sample Sources (%)			p-value

	Imported	Local	Fresh	
FOX				0.05 S
Intermediate	0 (0)	1 (7.7)	0 (0)	
Resistance	2 (15.4)	0 (0)	7 (53.8)	
Sensitive	0 (0)	2 (15.4)	1 (7.7)	
OX				0.7 NS
Intermediate	0 (0)	0 (0)	1 (7.7)	
Resistance	2 (15.4)	3 (23.1)	7 (53.8)	
Sensitive	0 (0)	0 (0)	0 (0)	
VAN				0.01 S
Intermediate	0 (0)	0 (0)	0 (0)	
Resistance	2 (15.4)	0 (0)	1 (7.7)	
Sensitive	0 (0)	3 (23.1)	7 (53.8)	
FA				0.7 NS
Intermediate	0 (0)	0 (0)	0 (0)	
Resistance	2 (15.4)	3 (23.1)	7 (53.8)	
Sensitive	0 (0)	0 (0)	1 (7.7)	
E				0.4 NS
Intermediate	0 (0)	0 (0)	2 (15.4)	
Resistance	2 (15.4)	3 (23.1)	6 (46.2)	
Sensitive	0 (0)	0 (0)	0 (0)	
T				0.08 NS
Intermediate	0 (0)	0 (0)	0 (0)	
Resistance	2 (15.4)	0 (0)	4 (30.8)	
Sensitive	0 (0)	3 (23.1)	4 (30.8)	
GM				0.7 NS
Intermediate	0 (0)	0 (0)	2 (15.4)	
Resistance	1 (7.7)	1 (7.7)	2 (15.4)	
Sensitive	1 (7.7)	2 (15.4)	4 (30.8)	
ENF				0.1 NS
Intermediate	0 (0)	0 (0)	1 (7.7)	
Resistance	2 (15.4)	0 (0)	3 (23.1)	
Sensitive	0 (0)	3 (23.1)	4 (30.8)	
OFL				0.5 NS
Intermediate	1 (7.7)	0 (0)	1 (7.7)	
Resistance	0 (0)	0 (0)	1 (7.7)	
Sensitive	1 (7.7)	3 (23.1)	6 (46.2)	

VAN=Vancomycin, FOX=Cefoxitin, OX=Oxacillin, FA=Fusidic acid, E=Erythromycin, T=Tetracycline, GM=Gentamicin, ENR=Enrofloxacin, OFL=Ofloxacin, MRSA=Methicillin-resistant *Staphylococcus aureus*

**Table-3:** ARP of MRSA isolates recovered from fresh and frozen chicken meat.

ARP	Number of antimicrobials	Number of antimicrobial classes	Number of isolates based on sample source			Total (%)

			Fresh	Local frozen	Imported frozen	
ME, FOX, OX, FA, E, T, GM, and ENF OFL; ME, VAN, FOX, OX, FA, E, T, GM, and ENF	9	6; 7	1	0	1	2 (15.4)
ME, VAN, FOX, OX, FA, E, T, ENF	8	6	0	0	1	1 (7.7)
ME, FOX, OX, FA, E, T, ENF	7	5	1	0	0	1 (7.7)
ME, VAN, FOX, OX, FA, E	6	4	1	0	0	1 (7.7)
ME, FOX, OX, FA, GM; ME, FOX, OX, FA, E; ME, FOX, E, T, ENF; ME, OX, FA, E, T; ME, OX, FA, E, GM	5	3; 4	4	1	0	5 (38.5)
ME, FOX, OX, FA; ME, OX, FA, E	4	2; 3	1	2	0	3 (23.1)
12	10		8	3	2	13 (100)

MRSA=Methicillin-resistant *Staphylococcus aureus*, ARP=Antimicrobial resistance patterns ME=Methicillin, FOX=Cefoxitin, OX=Oxacillin, FA=Fusidic acid, E=Erythromycin, T=Tetracycline, GM=Gentamicin, ENF=Enrofloxacin, VAN=Vancomycin

The results (Table-4) revealed that, after treatment with ozonated water (0.5 ppm/30 min/4°C), three samples (23.1%) were negative (no bacterial growth on the surface of the agar) and 10 samples (76.9%) were positive (>100 colonies/plate), while after an extended time to 45 min, nine samples (69.2%) were negative and four samples (30.8%) were positive (<30 colonies/plate). To evaluate the antibacterial efficacy of ozonated water (0.5 ppm) on reducing the MRSA count (log_10_ CFU/ml), four samples were selected (fresh first, fresh second, local frozen first, and imported frozen first), and the results showed that the bacterial counts before the treatments were 1.2×10^7^, 1.5×10^6^, 1.9×10^5^, and 1.8×10^5^ for fresh first, fresh second, local frozen first, and imported frozen first, respectively, whereas after treatments with ozonated water for 30 min, the levels of MRSA were decreased by 1-2 log_10_ CFU/ml, and this reduction was increased with an extension the exposure time to ozonated water for 45 min to reach 3-4 log_10_ CFU/ml (Table-5).

**Table-4:** Effect of ozonated water (0.5 ppm) treatment on MRSA isolates recovered from positive samples of fresh and frozen chicken meat.

Source	Total number of positive samples for MRSA before ozone treatment	Total number of negative samples for MRSA after ozone treatment (%)	

		30 min	45 min
Fresh	8	2 (25)	6 (75)
Local frozen	3	0 (0)	2 (66.7)
Imported frozen	2	1 (50)	1 (50)
Total	13	3	9
Efficiency (negative)		3/13 (23.1)	9/13 (69.2)

MRSA=Methicillin-resistant *Staphylococcus aureus*

**Table-5:** Antibacterial efficiency of ozonated water (0.5 ppm) on the reduction of MRSA count (log_10_ CFU/ml) from positive samples.

Sample code	log_10_ CFU/ml count before ozone treatment	log_10_ CFU/ml count after ozone treatment				

		30 min	log_10_ decreased	45 min	log_10_ decreased	Total log_10_ decreased
Fresh first	1.2×10^7^	1.1×10^5^	2	1.1×10^3^	2	4
Fresh second	1.5×10^6^	1.4×10^4^	2	1.4×10^2^	2	4
Local frozen first	1.9×10^5^	1.7×10^4^	1	1.7×10^3^	1	2
Imported frozen first	1.8×10^5^	1.6×10^3^	2	1.6×10^2^	1	3

MRSA=Methicillin-resistant *Staphylococcus aureus*, CFU=Colony-forming units

## Discussion

Contamination of meat with *S. aureus* can occur directly from infected animals destined for food production or can occur as a result of human contamination by poor hygiene procedures during production processes as humans can also harbor microorganisms [31]. Cross-contamination of chicken meat can also occur due to poor hygienic and storage conditions of these products [32]. This study was carried out to imply that MRSA contamination of chicken meat sold in the Wasit markets is not rare.

In this study, the prevalence of *S. aureus* in chicken meat was determined as 54.2%. This finding is similar to that obtained by Krupa *et al*. [33] who found that the prevalence of *S. aureus* in chicken meat is >52% and up to 93%. The prevalence of *S. aureus* in chicken meat samples of this study was higher than that of the USA (17.8%) [8], Egypt (21.4%) [34], and Romania (7.5%) [35].

The higher prevalence (59.4%) of *S. aureus* in fresh chicken meat during this study can be traced back to the fact that, in Iraq, most chickens are sold in plucking markets that lack hygienic measures which lead to increase the possibilities for the contamination of slaughtered chicken with *S. aureus*. Moreover, the relatively higher prevalence (55%) of *S. aureus* in local frozen chicken meat may be attributed to poor performance of slaughter operations which lead to increase the contamination potential. Furthermore, the results also revealed that 33.3% of *S. aureus* isolates were recognized as MRSA. This finding is similar to that reported by Dehkordi *et al*. [4]. In their study in Iran, the authors found that chicken meat had the highest prevalence of MRSA among other hospital food samples up to 32.43%. The prevalence of MRSA in *S. aureus* isolated from chicken meat samples of this research was higher than that of the USA (26%) [36], Canada (1.2%) [37], Germany (25.0%) [38], and Brazil (23.30%) [39]. A comparison between numerous studies can be difficult due to the wide variety of factors that contribute to the differences observed between the studies such as differences in culture methodologies and differences in the sampling techniques, and the type of sample (whole chicken or minced chicken meat) should also be considered.

The presence of resistant bacteria in chickens can lead to their presence in chicken carcasses and their products, which poses a risk to human health [40]. All MRSA isolates in this study showed a high prevalence of resistance to beta-lactams, FA, and E, while these isolates exhibited a low prevalence of resistance against T, ENF, GM, VAN, and OFL (Figure-1).

The increased resistance to beta-lactams (ME, OX, and FOX) could be related to the widespread usage of penicillin in livestock and poultry as feed additives and growth promoters, while resistance against macrolides could be attributed to application of spiramycin which is most commonly used for the promotion of growth in poultry production [41]; this use may support the selection of resistance to E in MRSA isolates due to cross-resistance relations among the chemically related antimicrobials. Resistance to tetracycline can be credited to its wide use in the prophylaxis and therapy of human and animal infections and as additives for livestock and poultry [42]. Moreover, the antimicrobial-resistant bacteria such as *Enterococci* spp. were detected in numerous food samples such as frozen broiler meat [42], and these bacteria exhibit resistance to several antibiotics such as bacitracin, ciprofloxacin, E, streptomycin, T, and VAN, which may be allocated the resistance to MRSA against these antimicrobials [43]. This finding is supported by the results recently obtained by Elmal and Can [44]. In their study in Turkey, they found that broiler meat was more commonly contaminated with vancomycin-resistant *Enterococci* among all other experienced food samples with the prevalence of 57.1%.

On the other hand, VAN was considered a magic drug for the treatment of resistant *S. aureus* to β-lactams, and this could be explained the significant effect (p=0.01) for VAN on MRSA isolates (Table-2). Resistance to fluoroquinolone among *S. aureus* isolates could be attributed to its use as prophylaxis in the poultry productions, which may have been selected for resistant strains to fluoroquinolones that exist today [45]. The onset of resistance to GM may be related to the use of apramycin (aminoglycoside, structurally related to GM) for veterinary treatment [46].

The finding of antibiotic resistance profile of this study is in accordance with the previous reports of Dehkordi *et al*. [4]. In their study in Iran, the authors found that the prevalence of antibiotic resistance in MRSA isolates against penicillin, ceftaroline, T, GM, E, ciprofloxacin, levofloxacin, and rifampicin was 100%, 100%, 100%, 80%, 80%, 40%, 40%, and 20%, respectively. Another study conducted by Waters *et al*. [36] in which they documented a high prevalence of resistance in *S. aureus* isolates recovered from meat and poultry products against β-lactams (penicillin and OX), T, fluoroquinolones, daptomycin, and VAN. Furthermore, Jackson *et al*. [47] reported that the prevalence of antibiotic resistance in MRSA isolated from beef meat against ciprofloxacin, E, gatifloxacin, levofloxacin, ceftriaxone, clindamycin, and T was 100%, 100%, 100%, 100%, 75%, 25%, and 25%, respectively.

The acquired results (Table-3) revealed that all MRSA isolates (100%) exhibited MDR to at least four antimicrobials. Multiple drug resistance has been defined as an isolate that displays resistance to three or more antimicrobials simultaneously [47]. Multi-resistance can be attributed to the presence of some resistance genes commonly found in *Staphylococci* that help to explain the spread of resistance to antibiotics [47]. In addition, MDR may reflect the acquisition of different elements of resistance in the same DNA molecule or unique elements, such as multidrug pumps, which specify the activity of the antibiotic efflux pumps incorporated in the membrane against numerous antimicrobial agents [48]. The mechanisms of genetic resistance could be chromosomal or plasmidic and represent a combination of endogenous and acquired genes [49].

MDR to at least three antimicrobials in MRSA isolated from retail meat was previously detectable by Jackson *et al*. [47] who found that all MRSA isolates exhibited MDR to at least three antimicrobials, and two classes of antibiotics of that 28.6 % of the isolates exhibited resistance to nine antimicrobials with the percentages of 14.3%, 14.3%, and 42.9% for the MDR model of AMP GEN PEN, AMP CIP ERY GAT PEN, and AMP CEF CIP ERY GAT LEV OXA PEN, respectively. Daka *et al*. [50] reported that the prevalence of MDR in MRSA isolated from milk samples to 3 or 4, 6, and 7 antimicrobials was 51%, 42.9%, and 6.1%, respectively. Furthermore, Momtaz *et al*. [51] found that 31.69% of the *S. aureus* isolates recovered from chicken meat were exhibited MDR against three or more antibiotics. Rong *et al*. [52] in their investigation found that 90.6% of the *S. aureus* isolates recovered from aquatic products displayed resistance to three or more antimicrobial agents.

O_3_ is used in a wide variety of agricultural products such as vegetables, fruits, and fish. It is characterized by a high oxidation potential that transmits bactericidal and viricidal possessions [15,53]. The results (Table-4) of the current study indicated that, after treatment with ozonated water for 30 min, 3/13 (23.1%) samples were negative for MRSA, while, after extension the exposure time for 45 min, the number of negative samples were increased to reach 9/13 (69.2%). In addition, the results (Table-5) revealed that, after treatment with ozonated water for 30 min, MRSA levels had decreased by 1-2 log_10_ CFU/ml, but this reduction increased with an extension the exposure time to 45 min to achieve 2-4 log_10_ CFU/ml, and this reduction is highly significant from a public health perspective.

O_3_ is a powerful wide-spectrum antimicrobial agent that is energetic against bacteria, fungi, viruses, protozoa, and bacterial and fungal spores [15]. The inactivation of bacteria by O_3_ can be related to its high instability that leads to its rapid decomposition in free radicals, so its reactivity is credited to the oxidizing power of these free radicals and their ability to spread through the biological membranes, attacking cellular components, disturbs usual cellular activity responsible for microbial damage [16-20,53]. The results of this study indicated that the number of persisting bacterial cells represented by CFU from treated samples was lower than that of untreated samples. In addition, the number decreased with the increased exposure time to ozonated water at the same concentration (0.5 ppm), and the plausible explanation may be due to longer O_3_ contact time with microorganisms which results in a reduction in the inactivation rate. Furthermore, O_3_ treatments were performed at 4°C. In general, a reduction in the temperature of the aqueous medium rises the solubility and stability of O_3_, enhancing its availability in the medium and consequently its efficacy rises [53].

The effectiveness of O_3_ treatment during immersion cooling as an intervention to improve the microbial safety of chicken carcasses had previously established by Jindal *et al*. [54] who found that O_3_ as 0.44-0.54 ppm at 4°C for 45 min reduced the levels of aerobic plate count, *Coliforms*, and *Escherichia coli* on broiler sticks by 95.5%. Furthermore, they stated that the microbial reductions after treatment for 15 min were lower than those acquired after treatment for 45 min, in which after O_3_ treatment for 15 min, the levels of *Coliforms* and *E. coli* were reduced by 1.50 and 0.01 log_10_, respectively, whereas after treatment for 45 min, the levels of *Coliforms* and *E. coli* were reduced to less than detection limit (0.00 log_10_ CFU/ml). Hecer *et al*. [55] in their study compared the effects of two antimicrobial applications (O_3_ and chlorine) as 1.5 ppm and 30 ppm for 7 min, respectively. They reported that the average effects of O_3_ and chlorine on the number of *Staphylococcus/Micrococcus* were 81.33% and 50%, respectively.

The results of this study are in accordance with the previous results of de Boer *et al*. [56] in which they described gaseous O_3_ as a successful intervention to destroy MRSA in extensively contaminated home environment when using O_3_ as an intervention to eliminate these bacteria from a carrier with eczema. Another study conducted by Burgassi *et al*. [57] estimated the bactericidal effect of O_3_ as 5-320 mg/L at 20°C for 15 min on *S. aureus*, MRSA, and *Pseudomonas aeruginosa*, and they found that no feasible bacteria of *S. aureus* and MRSA were detected after O_3_ treatment. In addition, Song *et al*. [58] in their study assessed the effectiveness and safety of topical O_3_ on the treatment of skin infection with MRSA, and they reported that ozonated oil could disinfect up to 98% of *S. aureus* and MRSA in 5 and 15 min, respectively. Furthermore, they concluded that ozonated water (1 mg/L) could sterilize 100% of *S. aureus* and MRSA in 1 min.

## Conclusion

From the data acquired from this study, it can be concluded that fresh and frozen chicken meat that sold in the dissimilar marketplaces of Wasit Province was extremely contaminated with *S. aureus* during the study period with a total prevalence of 54.2%; among those, 33.3% were recognized as MRSA. In addition, with respect to antibiotic resistance, the phenomenon of MDR was recognized in all experienced isolates whereby these isolates exhibited 12 ARPs to four or more antimicrobial agents (MDR), and then take into consideration that the contaminated food is an important vehicle for the transfer of resistance to antibiotics, so this prevalent of MDR is a public health concern when these life-saving antimicrobials are used to treat patients. Under the conditions designated in the existing study, O_3_ at the concentration of 0.5 ppm is highly active in reducing the number of MRSA-positive samples and the number decreased with increased contact time to ozonated water at the same concentration. These results are very important from the public health point of view. Moreover, these results have recommended that ozonated water can be used as a likely antibacterial intervention to disinfect broiler meat against pathogenic bacteria such as MRSA, both during immersion cooling in poultry slaughterhouses or before domestic cooking (at home and in restaurants).

## Authors’ Contributions

MHGK was responsible for all parts of this study (study design, samples collection, bacterial isolation and identification, O_3_ treatment, prepared the manuscript, data analysis, and corrections). The author read, finalized, and approved the manuscript.

## References

[1] Rodríguez-Lázaro D, Elena-Alexandra EA, Patricia PG, David GD, Fernández-Natal I, Dominguez-Gil M, Eiros-Bouza JM, Martin WM, Nicolau AI, Hernández M (2017). Detection and characterization of *Staphylococcus aureus* and Methicillin-resistant *S. aureus* in foods confiscated in EU borders. Front Microbiol.

[2] Peton V, Le Loir Y (2014). *Staphylococcus aureus* in veterinary medicine. Infect. Gen. Evol.

[3] Hennekinne JA, De Buyser ML, Dragacci S (2012). *Staphylococcus aureus* and its food poisoning toxins: Characterization and outbreak investigation. FEMS Microbiol. Rev.

[4] Dehkordi FS, Gandomi H, Basti AA, Misaghi A, Rahimi E (2017). Phenotypic and genotypic characterization of antibiotic resistance of methicillin resistant *Staphylococcus aureus* isolated from hospital food. Antimicrob. Resist. Infect. Control.

[5] Hasanpour DA, Khaji L, Sakhaei S.M.H, Mashak Z, Safarpoor DF, Safaree Y (2017). One-year prevalence of antimicrobial susceptibility pattern of Methicillin-resistant *Staphylococcus aureus* recovered from raw meat. Trop. Biomed.

[6] Hasman H, Moodley A, Guardabassi L, Stegger M, Skov RL, Aarestrup FM (2010). Spa type distribution in *Staphylococcus aureus* originating from pigs, cattle and poultry. Vet. Microbiol.

[7] Verhegghe M, Crombé F, Luyckx K, Haesebrouck F, Butaye P, Herman L (2016). Prevalence and genetic diversity of livestock-associated Methicillin-resistant *Staphylococcus aureus* on Belgian pork. J. Food Prot.

[8] Hanson BM, Dressler AE, Harper AL, Scheibel RP, Wardyn SE, Roberts LK, Kroeger JS, Smith TC (2011). Prevalence of *Staphylococcus aureus* and Methicillin-resistant *Staphylococcus aureus*(MRSA) on retail meat in Iowa. J. Infect. Public Health.

[9] Vandendriessche S, Vanderhaeghen W, Larsen J, Mendonca R, Hallin M, Butaye P, Hermans K, Haesebrouck F, Denis O (2014). High genetic diversity of methicillin-susceptible *Staphylococcus aureus*(MSSA) from humans and animals on livestock farms and presence of SCCmecremnant DNA in MSSA CC398. J. Antimicrob. Chemother.

[10] Smith TC, Pearson N (2011). The emergence of *Staphylococcus aureus* ST398. Vector Borne Zoonotic Dis.

[11] Zhang H, Wu J, Guo X (2016). Effects of antimicrobial and antioxidant activities of spice extracts on raw chicken meat quality. Food Sci. Hum. Wellness.

[12] EL-Dahshan HA, Hafez TA, EL-Ghayaty HA (2013). Effect of ozone on preservation of chilled chicken. Assiut Vet. Med. J.

[13] Muhlisin YC, Ji HC, Tae-Wook H, Sung K (2015). Bacterial counts and oxidative properties of chicken breast inoculated with *salmonella typhimurium* exposed to gaseous ozone. J. Food Saf.

[14] Kronn TG (2013). Non Thermal Plasma Treatment of Packaged Broiler Breast Fillets to Reduce Natural microflora and *Campylobacter jejuni*. MSC Thesis College of Science. University of Georgia.

[15] Khadre MA, Yousef AE, Kim JG (2001). Microbiological aspects of ozone applications in food: A review. J. Food Sci.

[16] Cardenas FC, Andres S, Giannuzi L, Zaritzky N (2011). Antimicrobial action and effects on beef quality attributes of a gaseous ozone treatment at refrigeration temperatures. Food Control.

[17] Muhlisin D, Utama T, Lee JH, Choi JH, Lee SK (2016). Effects of gaseous ozone exposure on bacterial counts and oxidative properties in chicken and duck breast meat. Korean J. Food Sci. Anim. Resour.

[18] Mahapatra AK, Muthukumarappan K, Julson JL (2005). Applications of ozone, bacteriocins and irradiation in food processing: A review. Crit. Rev. Food Sci. Nutr.

[19] Kim JG, Yousef A, Chism G (1999). Use of ozone to inactivate microorganisms on lettuce. J. Food Saf.

[20] Restaino L, Erampton EW, Hemphill JB (1995). Efficacy of ozonated water against various food-related microorganisms. Appl. Environ. Microbiol.

[21] Gabler FM, Smilanick JL, Mansour MF, Karaca H (2010). Influence of fumigation with high concentrations of ozone gas on post-harvest gray mold and fungicide residues on table grapes. Postharvest Biol. Technol.

[22] International Standardization Organization (ISO) 6888-1 (1999). Microbiology of Food and Animal Feeding Stuffs - Horizontal Method for the Enumeration of Coagulase Positive *Staphylococci* (*Staphylococcus aureus* and other species)-Part 1: Technique using Baird-Parker Agar medium.

[23] Food and Drug Administration (FDA) (2000). *Staphylococcus aureus* In: Bad Bug Book. Foodborne Pathogenic Microorganisms and Natural Toxins Handbook. Ch. 3.

[24] Bacteriological Analytical Manual (BAM) (2013). *Staphylococcus aureus*. Ch. 12.

[25] Kanaan M.H.G, AL-Shammary AHA (2013). Detection of methicillin or multidrug-resistant *Staphylococcus aureus*(MRSA) in locally produced raw milk and soft cheese in Baghdad markets. Iraqi J. Vet. Med.

[26] Fijałkowski K, Peitler D, Karakulska J (2016). *Staphylococci* isolated from ready-to-eat meat - Identification, antibiotic resistance and toxin gene profile. Int. J. Food Microbiol.

[27] Oxoid-Remel (2016). Laboratory Manual for Media and Diagnostic Kits.

[28] Clinical and Laboratory Standards Institute (CLSI) (2015). Performance Standards for Antimicrobial Susceptibility Testing;Twenty-Fifth Informational Supplement. CLSI Document M100-S25.

[29] Abdulateef ZA (2017). Assessment the availability of ozonated water on the bioavailability of deltamethrin in treated sheep's meat. Int. J. Adv. Res. Biol. Sci.

[30] Miles AA, Misra SS (1938). The estimation of the bactericidal power of the blood. J. Hyg.

[31] Sergelidis D, Angelidis AS (2017). Methicillin-resistant *Staphylococcus aureus*: A controversial food-borne pathogen. Lett. Appl. Microbiol.

[32] Wendlandt S, Schwarz S, Silley P (2013). Methicillin-resistant *Staphylococcus aureus*: A foodborne pathogen?. Annu. Rev. Food Sci. Technol.

[33] Krupa P, Bystroń J, Bania J, Podkowik M, Empel J, Mroczkowska A (2014). Genotypes and oxacillin resistance of *Staphylococcus aureus* from chicken and chicken meat in Poland. J. Poult. Sci.

[34] Osman K, Badr J, Al-Maary KS, Moussa I.M.I, Hessain A, Amin G.Z.M.S, Abo-Shama UH, Orabi A, Saad S (2016). Prevalence of the antibiotic resistance genes in coagulase-positive and negative-*Staphylococcus* in chicken meat retailed to consumers. Front. Microbiol.

[35] Ciolacu L, Stessl B, Bolocan AS, Oniciuc EA, Wagner M, Rychli K (2016). Tracking foodborne pathogenic bacteria in raw and ready-to-eat food illegally sold at the Eastern EU border. Foodborne Pathog. Dis.

[36] Waters AE, Contente-Cuomo T, Buchhagen J, Liu CM, Watson L, Pearce K, Foster JT, Bowers J, Driebe EM, Engelthaler DM, Keim PS, Price LB (2011). Multidrug-resistant *Staphylococcus aureus* in US meat and poultry. Clin. Infect. Dis.

[37] Weese JS, Avery BP, Reid-Smith RJ (2010). Detection and quantification of Methicillin-resistant *Staphylococcus aureus*(MRSA) clones in retail meat products. Lett. Appl. Microbiol.

[38] Feßler AT, Kadlec K, Hassel M, Hauschild T, Eidam C, Ehricht R, Monecke S, Schwarz S (2011). Characterization of Methicillin-resistant *Staphylococcus aureus* isolates from food and food products of poultry origin in Germany. Appl. Environ. Microbiol.

[39] Costa W.L.R, Ferreira JS, Carvalho JS, Cerqueira ES, Oliveira LC, Almeida R.C.D.C (2015). Methicillin-resistant *Staphylococcus aureus* in raw meats and prepared foods in public hospitals in Salvador, Bahia. Braz. J. Food Sci.

[40] Centers for Diseases Control and Prevention (CDC) (2013). Antibiotic Resistance Threats in the United States.

[41] Ventola CL (2015). The antibiotic resistance crisis: Part 1: Causes and threats. Pharm. Ther.

[42] Chantziaras I, Boyen F, Callens B, Dewulf J (2014). Correlation between veterinary antimicrobial use and antimicrobial resistance in food-producing animals: A report on seven countries. J. Antimicrob. Chemother.

[43] Santestevan NA, Zvoboda DA, Prıchula J, Pereıra RI, Wachholz GR, Cardoso LA, de Moura TM, Medeıros AW, de Amorın DB, Tavares M, d'Azevedo PA, Franco AC, Frazzon J, Frazzon APG (2015). Antimicrobial resistance and virulence factor gene profiles of *Enterococcus* spp. Isolates from wild *Arctocephalus australis*(South American fur seal) and *Arctocephalus tropicalis* (Subantarctic fur seal). World J. Microbiol. Biotechnol.

[44] Elmal M, Can HY (2018). The prevalence, vancomycin resistance and virulence gene profiles of *Enterococcus* species recovered from different foods of animal origin. Vet. Arhiv.

[45] Nelson JM, Chiller TM, Powers JH, Angulo FJ (2007). Fluoroquinolone-resistant *Campylobacter* species and the withdrawal of fluoroquinolones from use in poultry: A public health success story. Clin. Infect. Dis.

[46] Saenz Y, Zarazaga M, Lantero M, Gastanares MJ, Baquero F, Torres C (2000). Antibiotic resistance in *Campylobacter* strains isolated from animals, foods, and humans in Spain in 1997-1998. Antimicrob. Agents Chemother.

[47] Jackson CR, Davis JA, Barrett JB (2013). Prevalence and characterization of Methicillin-resistant *Staphylococcus aureus* isolates from retail meat and humans in Georgia. J. Clin. Microbiol.

[48] Kanaan MHG (2013). Isolation and Identification of Methicillin-resistant *Staphylococcus aureus*(MRSA) in Locally Produced Raw Milk and White Soft Cheese in Baghdad Province. MSc Thesis College of Veterinary Medicine, University of Baghdad.

[49] Levy SB (2002). Factors impacting on the problem of antibiotic resistance. J. Antimicrob. Chemother.

[50] Daka D, Silassie S, Yihdego D (2012). Antibiotic-resistance *Staphylococcus aureus* isolated from cow's milk in the Hawassa area, South Ethiopia. Ann. Clin. Microbiol. Antimicrob.

[51] Momtaz H, Dehkordi FS, Rahimi E, Asgarifar A, Momeni M (2013). Virulence genes and antimicrobial resistance profiles of *Staphylococcus aureus* isolated from chicken meat in Isfahan province, Iran. J. Appl. Poult. Res.

[52] Rong D, Wu Q, Xu M, Zhang J, Yu S (2017). Prevalence, virulence genes, antimicrobial susceptibility, and genetic diversity of *Staphylococcus aureus* from retail aquatic products in China. Front. Microbiol.

[53] Patil S (2010). Efficacy of Ozone and Ultrasound for Microbial Reduction in Fruit Juice. Doctoral Dissertation. College of Sciences and Health.

[54] Jindal V, Waldrou AL, Forsythe RH, Miller MJ (1995). Ozone and improvement of quality and shelf life of poultry producer. J. Appl. Poult. Res.

[55] Hecer C, Balci F, Udum CD (2007). The effects of ozone and chlorine applications on microbiological quality of chickens during processing. J. Biol. Environ. Sci.

[56] de Boer EL, van Elzelingen-Dekker CM, van Rheenen-Verberg MF, Spanjaard LMD (2006). Use of gaseous ozone for eradication of Methicillin-resistant *Staphylococcus aureus* from the home environment of a colonized hospital employee. Infect. Control Hosp. Epidemiol.

[57] Burgassi S, Zanardi I, Travagli V, Montomoli E, Bocci V (2009). How much ozone bactericidal activity is compromised by plasma components?. J. Appl. Microbiol.

[58] Song M, Zeng Q, Xiang Y, Gao L, Huang J, Jinhua HJ, Wu K, Lu J (2018). The antibacterial effect of topical ozone on the treatment of MRSA skin infection. Mol. Med. Rep.

